# Serum biochemistry of *Trachemys scripta elegans* and *Trachemys dorbignyi* (*Testudines*: Emydidae) bred in captivity in the Northeastern semiarid region of Brazil

**DOI:** 10.14202/vetworld.2020.1083-1090

**Published:** 2020-06-13

**Authors:** A. Gradela, V. N. Souza, M. M. Queiroz, A. C. Constantino, M. D. Faria, I. C. Pires, F. M. Correa

**Affiliations:** 1 Collegiate of Veterinary Medicine, Universidade Federal do Vale do São Francisco, Petrolina, Pernambuco, Brazil; 2 ALPHA Veterinary Clinical Analysis Laboratory, Petrolina, Pernambuco, Brazil; 3 Department of Statistics, Rhodes University, Grahamstown, South Africa

**Keywords:** albumin, biometry, creatinine, glucose, urea

## Abstract

**Aim:**

This study aimed to assess fundamental biochemical values of healthy animals and to provide useful data on comparative physiologies of *Testudines*, being assessed the serum biochemistry profiles, and body and tail biometry of *Trachemys scripta elegans* and *Trachemys dorbignyi* bred in interaction in the semiarid region of the São Francisco river valley.

**Materials and Methods:**

Serum biochemistry variables (urea, creatinine, glucose, total serum protein, albumin, globulin, and albumin/globulin ratio), and biometry values of the body (mass [body mass (BM)], maximum curvilinear length [carapace length (CL)], and width [carapace width (CW)] of the carapace, maximum curvilinear length [plastron length (PL)], and width [plastron width PW] of the plastron), and the tail (total length of the tail [TLT], pre-cloacal tail length [PrCL], post-cloacal tail length [PoCL]) were measured after 24 h fasting.

**Results:**

*T. s. elegans* displayed higher BM, CL, CW, PL, PW, AST, TP, albumin, and globulin values. *T. dorbignyi* displayed higher values of glucose, TLT, and PrCL. Variables aspartate aminotransferase (AST) and total protein (TP) in *T. s. elegans* and glucose in *T. dorbignyi* explained most of the variance between the species and could serve to distinguish them.

**Conclusion:**

We conclude that most of the differences between *T. s. elegans* and *T. dorbignyi* shall be explained by biometric variables, AST, TP, and glucose, which characterize interspecific differences. Our results point out terms of reference for these species bred in captivity in the semiarid region of Brazilian Northeastern region and serve as a model for the comparative intra- and inter-species physiology and as a base for the health assessment of these species.

## Introduction

*Trachemys scripta elegans* is a native species from the south of USA, and Northern Mexico. In Brazil, it is considered as an exotic and invading species, being, at the same time, the most frequently commercialized as a “pet.” The illegal dissemination of the species is a problem that has been described in many countries, which includes it at the top of the 100 worst invasive species in the world [1]. *Trachemys dorbignyi* is one among the native Brazilian species that may be threatened by *T. s. elegans*. It is a small sized chelonian, which may reach up to 25.0 cm of the carapace [2]. It is naturally distributed in the state of Rio Grande do Sul but may also be present in the Northeastern region of Argentina and Uruguay [3], and other Brazilian states due to the Illegal traffic of these animals and the abandonment by the owners increased the possibility of both species to share the same habitat.

Serum biochemistry shall be a useful tool to assess the turtle’s health [4]. The knowledge of the physiological serum biochemistry is crucial for the maintenance of these animals in captivity, or as “pets,” the rehabilitation for free-living, studies in extinction endangered species, monitoring of diseased animals [5], to assess the response to treatments, and for the diagnosis and prevention of diseases [6]. Serum biochemistry also represents a relevant tool for the monitoring of the metabolic rate, serving as an indicator of energetic, protein and mineral metabolism, and providing main elements for the interpretation of kidney, liver, bone, muscle, and pancreatic functionality. Age, size of the body, sex, year season, health condition, and diet may affect biochemical parameters and hinder the establishment of serologic values of reference, and the comparison among individuals and populations [7]. The hepatic functionality of reptiles is deemed to be similar to that of mammals and birds [8]. For this reason, bibliography suggests, the biochemical profile should be composed by total serum protein (total protein [TP]), glucose, uric acid, cholesterol, alkaline phosphatase; creatinine kinase, calcium, and phosphorus among the others [9]. Aspartate aminotransferase (AST) and alanine aminotransferase (ALT) also represent fundamental analyses for the diagnosis of liver diseases, as they provide help to assess alterations to the liver and bile ducts functionalities [10].

In view of the scarcity of studies on serum biochemistry in *Testudines* and the lack of studies in *T. s. elegans* and *T. dorbignyi*, we aimed to assess serum biochemical profiles of these species when bred in captivity in the Northeast Brazilian region in a situation of population interaction. With this work, we hope to contribute to the establishment of reference parameters and with to interspecific comparative studies.

## Materials and Methods

### Ethical approval

This research was carried out following standard operational procedure by SISBIO/IBAMA (protocol n° 38601-1), and approved by the ethical committee for studies and researches of the UNIVASF (protocol n° 0001/130314). Maintenance (No. 131/2013) and transportation (Process SMA/DeFau No. 13461/2012) licenses have been issued by IBAMA – Brazilian Institute of Environment and Renewable Natural Resources (Process SMA/DeFau No. 13461/2012).

### Study period

This study was carried out from November 2014 to October and 2015 in the Laboratory of Anatomy of Domestic and Wild Animals of the Agricultural Sciences Campus of the Federal University of Vale do São Francisco in Petrolina, Pernambuco, Brazil and at ALPHA Veterinary Clinical Analysis Laboratory, Petrolina-PE, Brazil.

### Animals

Specimen of *T. s. elegans* (n=28) and *T. dorbignyi* (n=22) from the Wild Animals Triage Center (CETAS) of the Tietê Ecological Park, Guarulhos (State of São Paulo) (23°29’23.15”S and 46°31’10.90”W) was carried to the Campus of Agricultural Sciences of the Federal University of the San Francisco Valley (UNIVASF), at Petrolina (State of Pernambuco) (9°23’34”S and 40°30’28”W), where the animals were bred in captivity under the condition of population interaction.

The transport was performed inside a closed truck. The animals were placed in cages at low carriage density, providing abundant space for movements and ventilation, to reduce stress, injuries, and deaths. During the travel, the animals were wet every 6 h and fed with vegetables. After 48 h of travel, the animals were received in good health conditions and without any mutilation at the UNIVASF. The turtles were unloaded in the UNIVASF with as less noise and movement as possible and placed in a paludarium with a maximum water depth of 25 cm, direct sunlight access and fed using industrialized feeds provided once a day. Total water substitution and enclosure cleaning were performed every 48 h. To avoid the proliferation of microorganisms, the water and the enclosure were treated using a water solution of methylene blue.

### Data collection

After a 120-day long adaptation to captivity, the animals having proper physical conditions, the absence of tumors, cutaneous lesions, or deformities of carapace and plastron were randomly selected and left in 48 h fasting. Afterward, they were taken to the Laboratory of Anatomy of Domestic and Wild Animals of UNIVASF for blood draws. For that, animals were contained with both hands placed on either side of the body and with the aid of a small gauze fragment, the thoracic limbs were pulled caudally and the head cranially so that the supra-occipital venous sinus was bilaterally distended with blood. Next, a blood collection needle was inserted 0.5 or 1.0 cm from the cervical midline, according to the size (smaller or larger) of the animal, at the midpoint of this line. Then, approximately 2.5 mL of blood was collected using 5.0 mL syringes and disposable needles (25×7) and deposited in a non-anticoagulant tube to obtain serum after centrifugation at 1200 G for 10 min in an Excelsa Baby centrifuge (Fanem, model 208N). The serum was frozen at −20°C until the performance of analyses.

After blood sampling, the animals had the body biometry and the secondary sexual characteristics biometry evaluated. The body characters that were assessed were: Body mass (BM), length (carapace length [CL]) and width (carapace width [CW]) maximums of carapace; and length (plastron length [PL]) and width (plastron width [PW]) maximums of plastron (Malvasio *et al*. 1999). The secondary sexual characteristics that were assessed were: Measurement of the linear length from the base of the tail to the cloacal orifice (pre-cloacal tail length [PrCL]), the linear length from the cloacal orifice to the extremity of the tail (post-cloacal tail length [PoCL]), and the total tail length (total length of the tail [TLT]=PrCL+PoCL). BM was assessed using an analytical precision balance (Bioprecisa^®^, Labmais Ltda., Curitiba, PR – Brazil), the body parameters using a measuring tape, and secondary sexual characters using a millimetric scale caliper.

### Biochemical analysis

The biochemical analyses were carried using commercial kits (Labtest^®^; Lagoa Santa, Minas Gerais – MG, Brazil) and included aspartate transaminase (AST), alanine transaminase (ALT), creatinine, urea, glucose, TP, total globulin, albumin, and albumin to globulin ratio (A/G ratio). The TP was measured by the modified biuret method and spectrophotometric reading at 550 nm. Albumin was determined by the modified bromocresol green method and spectrophotometric reading at 630 nm. Total globulin value was calculated by the difference between the values of these variables and, after this, the A/G ratio was calculated. Serum activities of AST and ALT were obtained by the modified Bowers & McComb method and UV/IFCC Kit for determination of total creatine kinase in serum sample, with spectrophotometric reading at 340 and 405 nm, respectively. Creatinine was assessed by end-point reaction using the enzymatic method and spectrophotometric reading at 546 nm. The glucose concentration was measured using the glucose liquiform^®^ commercial kit (reference n° 133, Labtest^®^; Lagoa Santa, Minas Gerais – MG, Brazil).

### Statistical analysis

Descriptive analyses of the mean±standard error (SE) were performed. Student’s t-test (a=5%) was used to assess the differences among the results of the samples for the studied species. The F test to identify if the variances are homogeneous was performed before Student’s t-test. To establish the limits of each species for biochemical parameters, confidence intervals were constructed for the averages (a=5%). BiPlot analysis with principal components was used to determine which biochemical parameters are responsible for the greater explanation of the variance found, and thus to be able to differentiate the species. A radar chart was drawn to describe the biochemical profiles of the species graphically. Statistical analyses were performed using the software R 3.3.1. (available in www.cran.r-project.org).

## Results

The F test indicated that all the variances of the analyzed samples were homogeneous. For the biochemical analyses, the number of samples available for each specie, descriptive statistics (means), confidence intervals, and differences between groups is reported in Table-1. AST, TP, albumin, and globulin were higher in *T. s. elegans* than in *T. dorbignyi*, which displayed higher glucose levels. Confidence intervals were significant for all analyzed chemical features; because between the limits of the confidence interval, the relative risk value 1.0 was not present.

**Table-1:** Means values±confidence intervals (95%) for biochemical parameters for *T. s. elegans* and *T. dorbignyi* bred in captivity in Petrolina (PE).

Biochemical parameters	Species	

	*T. s. elegans*	*T. dorbignyi*
AST (U/L)	232.3 (190.3; 274.4)^a^	32.6 (18.4; 46.8)^b^
ALT (U/L)	26.9 (13.7; 40.2)^a^	35.6 (21.2; 50.0)^a^
Urea (mg/dL)	30.7 (25.8; 35.7)^a^	28.1 (24.2; 32.1)^a^
Creatinine (mg/dL)	0.4 (0.3; 0.5)^a^	0.3 (0.3; 0.4)^a^
TP (g/dL)	5.6 (5.3; 6.0)^a^	4.2 (3.7; 4.7)^b^
Albumin (g/dL)	1.7 (1.4; 1.9)^a^	1.2 (0.9; 1.5)^b^
Globulin (g/dL)	3.8 (3.4; 4.3)^a^	2.9 (2.4; 3.5)^b^
A/G ratio	0.57 (0.4; 0.8)^a^	0.6 (0.4; 0.8)^a^
Glucose (mg/dL)	108.3 (92.0; 124.6)^b^	190.3 (151.5; 229.1)^a^

Means followed by the same letter in the same line do not differ significantly according to t-test at 5% probability. AST=Aspartate aminotransferase, ALT=Alanine aminotransferase, TP=Total serum protein, A/G ratio=Ratio between albumin/globulin

*T. s. elegans* displayed a larger size (p<0.05) in comparison with *T. dorbignyi*. In this last species, only the parameters TLT and PrCL were higher (p<0.05) than in the former. PoCL displayed no significant differences between the species (Table-2).

**Table-2:** Mean values±confidence intervals (95%) for body and tail biometrics of the turtle *T. s. elegans* and *T. dorbignyi* bred in captivity in Petrolina, PE.

Species	BM	CL	CW	PL	PW	TLT	PrCL	PoCL
*T. s. e.*	1462.6±479.3^a^	20.8±1.0^a^	17.1±0.5^a^	19.7±0.7^a^	13.3±0.4^a^	4.8±0.4^b^	2.0±0.3^b^	2.8±0.2^a^
*T. d.*	869.5±412.4^b^	18.8±0.9^b^	14.6±0.7^b^	16.9±0.9^b^	11.3±0.6^b^	5.6±0.5^a^	3.1±0.3^a^	2.7±0.2^a^

Different letters in the same column are significantly different by the t-test at 5% level. *T. s. e.*=*Trachemys scripta elegans, T. d.*=*Trachemys dorbignyi,* BM=Mass of the body *,* CL=Maximum curvilinear length of the carapace, CW=Maximum curvilinear width of the carapace, PL=Maximum curvilinear length of the plastron, PW=Maximum curvilinear width of the plastron, TLT=Total length of the tail, PrCL=Pre-cloacal length, PoCL=Post-cloacal length

In *T. s. elegans*, we observed a unimodal distribution of CL values at 20.77 cm and in *T. dorbignyi* at 18.79 cm. Frequency distribution for the maximum CL indicates that for *T. dorbignyi* more than 50% of the specimens have CL below 21.0 cm, whereas for *T. s. elegans*, the more than 50% of specimens have CL above 18.0 cm.

In the specie *T. s. elegans*, the biochemical parameters AST and ALT were negatively correlated with TLT and PoCL; urea was positively correlated with TLT and PrCL and albumin negatively correlated with TLT. In *T. dorbignyi*, there was a positive correlation between urea and PrCL and between creatinine and CL, CW, PL, PW, PoCL, and BM, and a negative correlation between glucose and CW, PL, PW, and PoCL (Table-3).

**Table-3:** Pearson correlation between biometric and biochemical parameters in *T. s. elegans* *(T. s. e.)* and *T. dorbignyi* *(T. d.)* turtles bred in captivity in Petrolina (PE).

*T. s. e.*	AST	ALT	Urea	Creatinine	TP	Albumin	Globulin	A/G ratio	Glucose
CL	−0.1.	0.1	0.3	−0.0	0.00	−0.17	0.3	−0.6	0.2
CW	−0.2	0.1	0.0	−0.3	−0.1	−0.4	−0.0	−0.0	−0.0
PL	−0.1	0.1	0.2	−0.2	−0.13	−0.3	0.1	−0.2	0.1
PW	−0.2	−0.0	0.1	−0.2	−0.05	−0.1	−0.0	−0.1	0.1
TLT	−0.4*	−0.5*	0.4*	0.1	−0.29	−0.5*	0.1	−0.2	0.2
PrCL	−0.3	−0.3	0.4*	0.1	−0.24	−0.3	−0.0	−0.1	0.2
PoCL	−0.4*	−0.4*	0.0	0.1	−0.23	−0.4	0.0	−0.1	0.1
BM	−0.0	0.2	0.5*	−0.2	0.13	−0.2	0.3	−0.2	−0.0

***T. d.***	**AST**	**ALT**	**Urea**	**Creatinine**	**TP**	**Albumin**	**Globulin**	**A/G ratio**	**Glucose**

CL	0.3	0.3	−0.4	0.5*	0.1	0.3	−0.1	0.2	−0.4
CW	0.4	0.3	−0.3	0.6*	0.1	0.3	−0.1	0.2	−0.5*
PL	0.4	0.3	−0.4	0.5*	0.1	0.3	−0.1	0.1	−0.5*
PW	0.4	0.4	−0.4	0.5*	0.1	0.2	0.0	0.1	−0.5*
TLT	0.0	0.1	0.2	−0.2	0.0	−0.2	0.1	0.0	−0.3
PrCL	−0.2	−0.1	0.5*	−0.4	0.1	−0.2	0.2	−0.0	−0.0
PoCL	0.2	0.2	−0.1	0.5*	0.2	−0.0	0.2	−0.0	−0.5*
BM	0.3	0.3	−0.3	0.6*	0.0	0.2	−0.1	0.1	−0.3

* significant at 5% error probability. AST=Aspartate aminotransferase, ALT=Alanine aminotransferase, TP=Total serum protein, A/G ratio=Relation albumin/globulin BM=Mass of the body, CL=Maximum curvilinear length of the carapace, CW=Maximum curvilinear width of the carapace, PL=Maximum curvilinear length of the plastron, PW=Maximum curvilinear width of the plastron, TLT=Total length of the tail, PrCL=Pre-cloacal length, PoCL=Post-cloacal length

For *T. s. elegans*, there was a positive correlation between TP and ALT, TP and Globulin and between A/G ratio and albumin and a negative correlation between A/G ratio and globulin. For *T. dorbignyi*, there was a positive correlation between AST and ALT; PT and globulin, A/G ratio and albumin (Table-4).

**Table-4:** Pearson correlation among the biochemical parameters in *T. s. elegans (T. s. e.)* and T *. dorbignyi (T. d.)* turtles bred in captivity in Petrolina (PE).

*T. s. e.*	AST	ALT	Urea	Creatinine	TP	Albumin	Globulin	A/G ratio	Glucose
AST	----	----	----	----	----	----	----	----	----
ALT	0.3	----	----	----	----	----	----	----	----
Urea	0.0	0.0	----	----	----	----	----	----	----
Creatinine	−0.1	0.1	−0.0	----	----	----	----	----	----
TP	0.3	0.4*	0.1	−0.1	----	----	----	----	----
Albumin	0.1	0.1	−0.1	0.1	0.2	----	----	----	----
Globulin	0.2	0.3	0.2	−0.1	0.7*	−0.4	----	----	----
A/G ratio	−0.0	−0.1	−0.2	0.1	−0.2	0.6*	−0.8*	----	----
Glucose	−0.3	0.1	0.1	0.2	−0.1	0.3	−0.2	0.2	----

***T. d.***	**AST**	**ALT**	**Urea**	**Creatinine**	**TP**	**Albumin**	**Globulin**	**A/G ratio**	**Glucose**

AST	----	----	----	----	----	----	----	----	----
ALT	0.9*	----	----	----	----	----	----	----	----
Urea	−0.2	0.1	----	----	----	----	----	----	----
Creatinine	0.2	0.2	−0.1	----	----	----	----	----	----
TP	−0.2	−0.2	0.1	0.2	----	----	----	----	----
Albumin	−0.1	−0.1	0.1	0.4	−0.0	----	----	----	----
Globulin	−0.2	−0.2	0.0	0.0	0.9*	−0.5	----	----	----
A/G ratio	−0.2	−0.2	0.3	0.1	−0.0	0.8*	−0.4	----	----
Glucose	0.3	−0.3	−0.0	−0.3	−0.3	0.1	−0.3	0.2	----

* Significant at 5% error probability AST=Aspartate aminotransferase, ALT=Alanine aminotransferase, TP=Total serum protein, A/G ratio=Relation albumin/globulin

**Figure-1:**
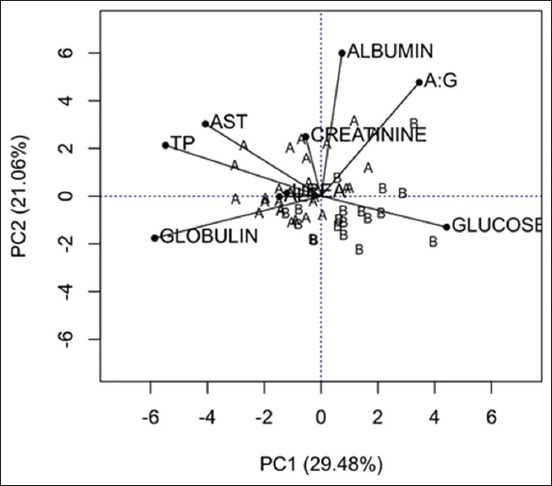
Principal component analysis to assess the relationship between the biochemical components and the species (A=*T. s. elegans*; and B=*T. dorbignyi*). AST: Aspartate aminotransferase; ALT: Alanine aminotransferase; TP: Total serum protein; A/G ratio: Ratio between albumin/globulin.

The PCA analysis suggested that most of the variation between the species could be explained by the biochemical variables: AST, TP, and glucose. *T. s. elegans* showed the highest levels of AST and TP, associated with lower glucose levels. *T. dorbignyi* displayed the lowest AST and TP values, but with higher levels of glucose (Figure-1). The PCA analysis suggested that most of the variation between the species could be explained by the biochemical variables: AST, TP, and glucose. T. s. elegans showed the highest levels of AST and TP, associated with lower glucose levels. T. dorbignyi displayed the lowest AST and TP values, but with higher levels of glucose (Figure-1). Radar chart of the biochemical profile of the studied species highlighted the importance of the variables that explained most of the variance found between species as a way of distinguishing them, being AST and PT in the *T. s. elegans* and glucose in *T. dorbignyi*.

## Discussion

Serum biochemistry assessment reflects the organic activity of the individual, showing the metabolic situation of its tissues. It enables the assessment of tissue lesions, malfunctioning of the internal organs, adaptations of the animals to nutritional or physiological variations, and specific metabolic failures; being very useful both for diagnosis and prognosis of diseases [11,12]. Biochemical parameters of reptile’s blood are not well defined and quoted in references, as in most of the domestic animals, since their health status depends directly on the captive management, being the type of diet and the inadequate environment, the main factors causing diseases [13]. In addition, factors that can cause changes in blood biochemical elements, such as age, size, sex, season, health, habitat, body temperature alterations, water availability, and reproductive cycle, among others, may cause variation of serum biochemistry. For these reasons, this study aimed to assess serum biochemistry values of turtles of the species *T. s. elegans* and *T. dorbignyi* raised in captivity in the Northeastern semi-arid region from Brazil in a situation of interaction between the species, seeking to establish reference parameters for both species.

The absence of injuries and/or adherence in the carapace, plastron, head, neck, tail, and free members suggested that turtles were clinically healthy. CL values [14] and tail length revealed that the specimen from both species was adults [14,15]. This fact occurred first because specimens are usually released by their owners into ponds, lakes, rivers and bodies of water, such as the Tietê Ecological Park, when they reach large body size and are no longer attractive as pets, and second, because adults have higher survival rates and lower mortality rates than juveniles [16].

*T. s. elegans* is considered a medium-sized species. It can reach a maximum length of 20.0-60.0 cm [17] and *T. dorbignyi* is considered a small-sized species [14]. These data corroborate with our findings, as we observed *T. s elegans* display a larger mean body size compared with *T. dorbignyi*. In *T. s. elegans* we observed a unimodal distribution of CL values. This distribution agrees with the results of the studies performed by [17,18]. The mean PL values (19.5±2.3 cm) indicated that all the specimens were sexually mature. In *T. s. elegans*, sexual maturity is achieved as the plastron reaches 9.0-10 cm of maximum length in males, and 17.4-19.3 cm in females [19].

The unimodal distribution at 18.79 cm observed in *T. dorbignyi* agreed with described in the previous works [14.15] that observed in adults values of 18.0 cm [15], or 19.0 cm [14] in males, and 20.0 cm [15] or 22.0 cm in females. In this species, sexual maturity is reached as CL reaches 13.0 cm in males, and 15.0-16.0 cm in females [14].

We suppose that regular supply and quality of food in captivity fostered the growth of *T. s elegans*, which displayed BM, CL, PL, and PW values higher than those described in specimens of this species captured in Brasilia (DF, Brazil) [20]. In contrast with these data, *T. dorbignyi* seems to have suffered from the competition with *T. s. elegans* in relation to food sources and perhaps even hunting pressure, since it presented BM, CL, CW, PL, and PW values similar to those of males of the same species bred in the extreme south of the state of Rio Grande do Sul [14]. These results can be explained because *T. s. elegans* presents great adaptive capacity and invasive power, needing only standing water, a place for sunbathing, and a climate without extreme variations for its adaptation, as provided during this study.

Analysis of the AST is not deemed to be a specific test, once it is found in all tissues in reptiles, with the highest concentration in liver and muscles. Its increase shall be observed in generalized diseases infectious and toxic hepatitis, biliary obstruction, cirrhosis and hepatic steatosis, hemolysis, selenium/Vitamin E deficiency, and intense muscular exercise [21]. In sea turtles, a range of 100-350 U/L has been suggested, and higher values may be suggestive of liver damage, in the skeletal muscle or in the myocardium [13]. The mean AST values in *T. s. elegans* were included in this interval, but they were higher than those of *Caretta caretta* [5,22], and lower than the *Chelonia mydas* [23]. These differences among the mean AST values have already been described between free-living and confined animals [23], and among heat-stressed animals [24], such as those used in this study. This is because in Petrolina, the maximum daily average temperature is above 33°C according to NASA’s Modern-Era Retrospective Analysis for Research and Applications 2. In *T. dorbignyi*, AST mean values were excluded of this interval and were significantly lower (p<0.05) than those in *T. s elegans*. As both species were housed in the same environment and submitted to the same handling and feeding, we shall infer, the lower AST values in *T. dorbignyi* are due to interspecific differences.

On the other side, ALT is a more specific marker of hepatic lesions. As being more concentrated in the hepatocytes than in other tissues, it is used in the control of hepatopathies, lung abscesses, and carbon compounds poisoning [12]. The variability between 10 and 30 UI/L was observed in reptiles. Our study highlighted, the mean ALT values did not show differences between the species and stayed inside the interval that has been described above only in *T. s. elegans*. It is believed that heat stress may have caused some tissue damage and increased the ALT levels [25] in *T. dorbignyi*. The AST/ALT ratio in this species ranged from 0.87 to 0.94, suggesting the presence of non-alcoholic steatohepatitis [26]. On the other hand, in *T. s. elegans* the AST/ALT ratio ranged from 6.82 to 13.89, which seems to be indicative of liver fibrosis [26]. As compared to the literature data, the mean values observed in both species were higher than in *C. caretta* [22], and lower than in *C. mydas* [23].

TLT and PoCL are relevant for the evaluation of sexual dimorphism in both studied species, being this measure in males higher and less, respectively, than in females [27]. These correlations have never been described before, as the literature only mentions correlations between ALT and BM, CL and CW [24].

Ureotelic species, such as reptiles, have the end product of nitrogen metabolism, uric acid instead of urea; thus, most reptiles present normal values for urea of <15 mg/dL [20]. However, in some terrestrial chelonians the values may be between 20 and 100 mg/dL, as observed in *T. s. elegans* and *T. dorbignyi*. High urea value shall be observed in case of high protein diet, or due to excretion renal rate or the conditions of the liver of the animals [13]. The mean urea value was like those described in *Kinosternon scorpioides* [28], but lower than those described in *C. caretta* [22] and *C. mydas* [23]. *T. s. elegans* displayed a positive correlation between the concentration of urea and BM, TLT, and PrCL. In *T. dorbignyi*, urea concentration was positively correlated only to PrCL. This datum had never been described earlier in the literature. PrCL measurement is usually higher in males to ease the accommodation of the penis in the cranial portion of the tail, and to benefit its exposition and introduction inside of the female cloaca.

*T. s. elegans* and *T. dorbignyi* had similar mean creatinine concentrations, and in both, the values were lower than 1.0 mg/dL, as expected in reptiles [20], having no meaning for the diagnosis of renal and pre-renal diseases [22]. The mean values observed in both species were slightly lower than those of *C. caretta* [5], and much lower than those of *C. mydas* [23]. In *T. dorbignyi*, we observed a positive correlation between creatinine concentration and muscular mass. This finding has been observed because plasma creatinine derived from the catabolism of the creatinine which is present in the muscular tissue, thus the concentration of plasma creatinine decreases in case of atrophy of the muscular tissue or associated diseases, or in case of prolonged or intense physical exercises. The significant positive correlation between creatinine and BM, CL, CW, PL, PW, and PoCL in *T. dorbignyi* allowed us to infer that the values of serum creatinine in females are higher than in males, as females of this species are larger [16] and have greater PoCL than males. In addition, these findings confirmed that *T. dorbignyi* were in competition with *T. s. elegans* for food sources and/or suffering hunting pressure for them.

Total serum protein (TP) values observed in both *T. s. elegans* and *T. dorbignyi* agree with the values considered normal in reptiles (3.0-8.0 mg/dL) [13]. As both species received the same diet and the same environmental conditions, we believe, the significantly higher values observed in *T. s. elegans* could be associated with interspecific differences. The mean TP values observed in *T. s. elegans* were like those of *K. scorpioides* [28] and higher than those of *C. caretta* [5,22]. *T. dorbignyi* presented mean values similar to those of *C. caretta* [5,22]. These differences could be explained by the species, different environmental conditions, and by the higher protein diet for animals bred in captivity [29].

The main function of albumin produced in the liver is to stabilize the level of fluids inside the vessels. In healthy reptiles and mammals, albumin represents the main protein fraction [20]. The average values of albumin were significantly higher in *T. s. elegans*, which presented concentrations slightly higher than those of *C. caretta* [5,22] and lower than those of *C. mydas* [23]. The concentration of albumin in *T. dorbignyi* was lower than that described in *C. caretta* [5,22] and higher than in *C. caretta* [4]. The literature attributes differences in serum albumin concentration to diet, size of animals, habitat and age [22]; however, the results of this study suggested that they were associated with specie and body size.

Globulin is determined from the difference between TP and albumin. When the concentration of globulin is high, it inhibits the synthesis of albumin in the liver to maintain the blood protein concentration constant, and, as a consequence, maintain the osmotic pressure constant. As observed with albumin and TP, the serum values of the globulin in *T. s. elegans* were significantly higher than in *T. dorbignyi*. These values were similar [5] or higher [22] than those of *C. caretta* and lower than those of *C mydas* [23]. In both *T. s. elegans* and in *T. dorbignyi*, globulin was higher than albumin so that the differences in the PT values reflected the variations occurred in this fraction, in agreement with previous findings [20]. The positive correlation between TP and globulin observed in both species confirmed this finding. As the animals were sexually matured, we believe the higher globulin/albumin ratio can be associated to the accumulation of natural antibodies, the gamma globulins, which happens during the development [29].

We observed no significant difference in A/G ratio between *T. s. elegans* and *T. dorbignyi*, which was similar to the results obtained in *C. caretta* [22], slightly higher than had been previously described in this species [5] and slightly lower than in *C. mydas* [23].

*T. dorbignyi* displayed mean glucose values significantly higher than those of *T. s. elegans*, and higher than those of *K. scorpioides* [28] and *C. caretta* [5]. Considering the confidence intervals, it can be stated with 95% accuracy that the lower and upper confidence limits observed in *T. s. elegans* (92.00; 124.57) and *T. dorbignyi* (151.54; 229.09) are representative of the general population of these species. The intervals we described in *T. s. elegans* are quite similar to those of *C. mydas* during rehabilitation (95.0; 145.0) [23]. The serum level of glucose in healthy animals shall vary according to the species, nutritional status, environmental conditions, stress, and physiological conditions, and in some species also due to seasonal variations [13]. Acute stress may alter glucose level in sea turtles [30,31] and values above 200 mg/dL are indicative of hyperglycemia and strong evidence of Type 2 diabetes mellitus (Type 2 DM) [32]. In this study, we observed glucose levels above 200 mg/dL in 41% (9/22) of *T. dorbignyi* (229.30-558.20 mg/dL), and in just 11% (3/28) of *T. s. elegans* (200.60-209.20 mg/dL). Similar ALT values in both the species ruled out the hypothesis of Type 2 DM, suggesting that the higher values of glucose we observed in *T. dorbignyi* could be associated with the differences in the metabolic response and stresses associated with the competition for foods, and hunting pressure caused by *T. s. elegans*.

**Figure-2:**
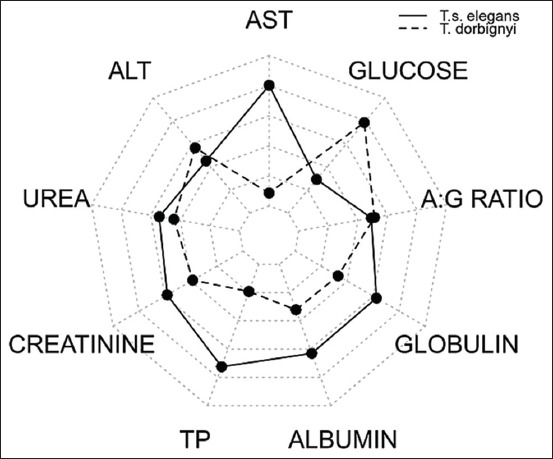
Radar chart of the biochemical profile of the studied species. AST: Aspartate aminotransferase; ALT: Alanine aminotransferase; TP: Total serum protein; A/G ratio: Ratio between albumin/globulin.

The biplot analysis of the main component (Figure-1) highlighted that the main biochemical variables that explained most of the variations between the two species were AST, TP, and glucose. In *T. s. elegans*, AST and TP values were higher, with lower glucose levels, and in *T. dorbignyi*, AST and TP were lower, with higher levels of glucose. In turn, the radar chart of the biochemical profile (Figure-2) highlighted the variables that distinguished the two species, which were AST and TP in *T. s. elegans* and glucose in *T. dorbignyi*.

## Conclusion

The results of this study culminated with the establishment of reference intervals of serum biochemical variables for the species *T. s. elegans* and *T. dorbignyi*. This work is the first one on biochemical parameters for these two species bred in captivity in the semiarid Northeastern region of Brazil in an interaction situation. The biochemical variables AST, TP, and glucose explained most of the variability between the two species: Higher levels of AST and TP in *T. s. elegans*, and of glucose in *T. dorbignyi*. These values were influenced by biometric differences, interspecific metabolic response and, in the case of glucose, also by the stress caused by the invasive power and hunting pressure caused by *T. s. elegans*. The results of this research may serve as a model for the comparative physiology inside and among species and may serve as a base of study for these species. These findings may, therefore, be used to identify biochemical disturbances and to follow physiologic adaptations required to the animals to face environmental changes.

## Authors’ Contributions

AG: Substantial contribution to the concept and design of the study; contribution to data collection, interpretation, and contribution to manuscript preparation and critical revision. VNS: Substantial contribution to data collection, and contribution to manuscript critical revision. MDF: Contribution to data collection and critical revision. MMQ; ACC; ICP: Substantial contribution to data collection and data analysis. FMC: Substantial contribution to the statistical data analysis and interpretation and critical revision. All authors read and approved the final manuscript.
